# Androgen Receptor is a Negative Regulator of PRDM16 in Beige Adipocyte

**DOI:** 10.1002/advs.202300070

**Published:** 2023-05-21

**Authors:** Shiting Zhao, Tao Nie, Lei Li, Qiaoyun Long, Ping Gu, Yuwei Zhang, Wei Sun, Zexin Lin, Qing Liu, Yue Qi, Wei Wang, Mengyuan Xie, Kerry Loomes, Chenleng Cai, Donghai Wu, Hannah Xiaoyan Hui

**Affiliations:** ^1^ CAS Key Laboratory of Regenerative Biology Guangdong Provincial Key Laboratory of Stem Cell and Regenerative Medicine Guangzhou Institutes of Biomedicine and Health Chinese Academy of Sciences Guangzhou 510530 China; ^2^ School of Biomedical Sciences The Chinese University of Hong Kong Hong Kong 999077 China; ^3^ GIBH‐CUHK Joint Research Laboratory on Stem Cell and Regenerative Medicine, Guangzhou Institutes of Biomedicine and Health Chinese Academy of Sciences Guangzhou 510530 China; ^4^ School of Basic Medicine Hubei University of Arts and Science Xiangyang 441053 China; ^5^ University of Chinese Academy of Sciences Beijing 100049 China; ^6^ Department of Endocrinology Jinling Hospital Nanjing University School of Medicine Nanjing 210016 China; ^7^ Joint School of Life Sciences Guangzhou Institutes of Biomedicine and Health Chinese Academy of Sciences Guangzhou Medical University Guangzhou 511436 China; ^8^ School of Biological Sciences and Maurice Wilkins Centre University of Auckland Auckland 1142 New Zealand; ^9^ China‐New Zealand Joint Laboratory on Biomedicine and Health Guangzhou 510530 China

**Keywords:** androgen, androgen receptor, beige adipocyte, PRDM16

## Abstract

PRDM16 (PR domain containing protein 16) serves as a dominant activator of brown and beige adipocyte. However, mechanisms underlying the regulation of PRDM16 expression are incompletely understood. A *Prdm16* luciferase knockin reporter mouse model is generated, enabling high throughput monitoring of *Prdm16* transcription. Single clonal analysis reveals high heterogeneity of *Prdm16* expression in the inguinal white adipose tissue (iWAT) cells. Amongst all transcription factors, androgen receptor (*Ar*) shows the strongest negative correlation with *Prdm16*. A sex dimorphism for *PRDM16* mRNA expression is present in human WAT, with female individuals exhibiting increased expression than males. Androgen‐AR signaling mobilization suppresses *Prdm16* expression, accompanied by attenuated beiging in beige adipocytes, but not in brown adipose tissue. The suppressive effect of androgens on beiging is abolished upon overexpression of *Prdm16*. Cleavage under targets and tagmentation mapping reveals direct binding of AR within the intronic region of *Prdm16* locus, whereas no direct binding is detected on *Ucp1* and other browning‐related genes. Adipocyte‐selective deletion of *Ar* potentiates beige cell biogenesis whereas adipocyte‐specific overexpression of *AR* attenuates white adipose beiging. This study highlights an essential role of AR in negative regulation of PRDM16 in WAT and provides an explanation for the observed sex difference in adipose beiging.

## Graphical abstract

Utilizing a PRDM16 reporter model, androgen receptor (AR) is identified as the strongest negative regulator of PRDM16, which displays a sex dimorphic expression in both human and mouse. Androgen‐AR complex binds at the intronic region of *Prdm16*, and thereby suppresses its expression and mitigates adipose browning. The study provides a new explanation for sex‐difference in adipose browning.

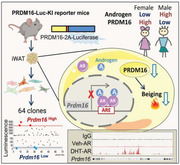

## 1 Introduction

Obesity poses a grave threat to health, contributing to increased risks associated with type 2 diabetes, fatty liver, cardiovascular disease and even COVID‐19.^[^ ^1^ ^]^ In the past decade, functional brown adipose tissue (BAT) has been detected in adult human and reignites interest in targeting thermogenic adipocytes as potential therapeutic strategies.^[^ ^2^ ^]^ Seminal work by Wu et al. identified thermogenic competent adipocytes scattered within white adipose tissue (WAT), termed beige adipocytes.^[^ ^3^ ^]^ Activation of brown and beige adipocytes improves body weight and adiposity, and confers additional metabolic benefits including insulin‐sensitization, improved lipid/glucose‐clearance, and anti‐atherosclerosis and anti‐cancer effects.^[^ ^4^ ^]^ Nevertheless, despite similar functionality, brown and beige adipocytes are of distinct cellular lineage and their mechanisms of action are also not identical.

PRDM16 (PR domain containing 16) was first identified as a master transcription regulator during a search for bona fide brown adipose identity factor.^[^ ^5^ ^]^ In brown adipocytes, PRDM16 serves as a cell fate determinator to switch between skeletal myoblasts and brown adipocytes.^[^ ^6^ ^]^ Later studies found that transgenic over‐expression of PRDM16 robustly induces beige adipocyte development in white adipose depots.^[^ ^7^ ^]^ By comparison, deletion of PRDM16 in mature adipocytes leads to complete ablation of beige fat function, and severe insulin resistance, with modest impact on BAT.^[^ ^8^ ^]^ These findings establish a key function for PRDM16 in beige adipocyte biogenesis.

Mechanistically, PRDM16 forms complexes with various transcriptional cofactors in a promoter‐dependent context, acting bifunctionally to turn on a full set of brown‐selective genes while repressing white adipocyte‐selective genes.^[^ ^5^, ^9^ ^]^ Intriguingly, PRDM16 also exerts metabolic‐protective functions in WAT beyond modulating adipose browning, including repression of adipose fibrosis and arborization of intra‐adipose sympathetic fibers.^[^ ^10^ ^]^

Nevertheless, in contrast to the understanding on its physiological functions, much less is known as to how PRDM16 expression, especially at the transcriptional level, is regulated. Using an in‐house generated luciferase‐based *Prdm16* knock‐in reporter mouse model and single clonal analysis, we found high cellular heterogeneity in *Prdm16* expression in stromal vascular cells (SVC) of mouse inguinal WAT (iWAT). Importantly, the androgen receptor (*Ar*) was identified as the transcription factor showing the strongest negative correlation with *Prdm16*. Consistently, a sex dimorphic pattern for *PRDM16* expression was observed in both human and mouse WAT. AR exerts an inhibitory effect on *Prdm16* transcription through its ligand‐dependent, genomic action by directly binding on the *Prdm16* locus. Furthermore, adipocyte‐selective deletion or overexpression of AR in mice either prompted or inhibited brown remodeling in WAT but not in BAT. These data highlight a pivotal role of androgen‐AR signaling in regulating PRDM16 expression and beiging in WAT.

## 2 Results

### 2.1 Generation of a PRDM16 Reporter Mouse Model

**Figure 1:**
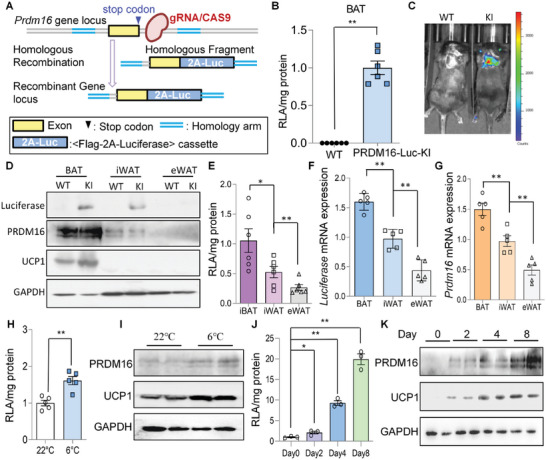
Establishment of a luciferase‐based PRDM16 reporter knock‐in mouse model. A) Schematic diagram of the strategy for generating PRDM16‐Luc KI mice (KI). B) Relative luciferase activity (RLA) in interscapular BAT. C57BL/6J mice were used as the wild‐type (WT) (*n* = 6/group). C) Live imaging of luminescence in WT and KI mice. D) Western blot of Luciferase, UCP1, and PRDM16 in WT and KI mice. E–G) RLA (E), qPCR analysis of Luciferase (F), and *Prdm16* mRNA expressions (G) in adipose tissues (*n* = 5/group). H,I) 8‐week‐old male PRDM16‐Luc KI mice were housed at room temperature or cold for 3 days and the luciferase activity (H) and protein levels of UCP1 and PRDM16 (I) in iWAT were measured (*n* = 5/group). J,K) RLA (J) and western blotting (K) in primary iWAT stromal vascular cells (SVC) from KI mice during the differentiation process (*n* = 3/group). Data are presented as mean ± SEM; statistical significances between groups were assessed by two‐sided unpaired Student's *t*‐test (B,H) and one‐way ANOVA (E,F,G,J); *p < 0.05, **p < 0.01.

To allow reliable and sensitive quantitation of endogenous PRDM16 gene expression, a reporter mouse with a *Prdm16* knock‐in allele (C57BL/6J‐*Prdm16*‐T2A‐Luc) was generated (termed PRDM16‐Luc KI mice). By CRISPR/Cas9‐mediated homologous recombination, open reading frame encoding T2A peptide and firefly luciferase (Luc) was positioned in frame before the stop codon of murine *Prdm16* gene (**Figure** **1**A); successful gene editing was verified by PCR and sequencing (data not shown).

Luciferase expression in PRDM16‐Luc KI reporter mice was examined by quantitation of luciferase activity in interscapular BAT (Figure 1B), in vivo and ex vivo luminescence imaging (Figure 1C and Figure S1A, Supporting Information), and western blotting (Figure 1D). Luciferase activity and its expression at both protein and mRNA levels were detected at the highest level in interscapular BAT, moderately in iWAT and lowest in epididymal white adipose tissue (eWAT) (Figure 1D–F). These analyses were in line with *Prdm16* mRNA expression as measured by qPCR (Figure 1G). Furthermore, the mRNA expressions of *Prdm16* and *Luciferase* exemplified strong correlation in all three adipose tissues (Figure S1B, Supporting Information). mRNA expression of *Ucp1* showed a similar trend (Figure S1C, Supporting Information). Luciferase activity was increased after cold exposure in mouse iWAT or in iWAT SVCs during in vitro adipogenic differentiation (Figure 1H,J), consistent with the induction of PRDM16 protein (Figure 1I,K). Collectively, these data demonstrate that the PRDM16‐Luc KI mouse is a reliable research tool where the magnitude of luciferase activity (PRDM16^LUC^) faithfully and robustly reports endogenous *Prdm16* transcription both in vivo and in vitro.

### 2.2 PRDM16 Is Heterogeneously Expressed in iWAT Cells and Inversely Associated with Androgen Receptor

**Figure 2:**
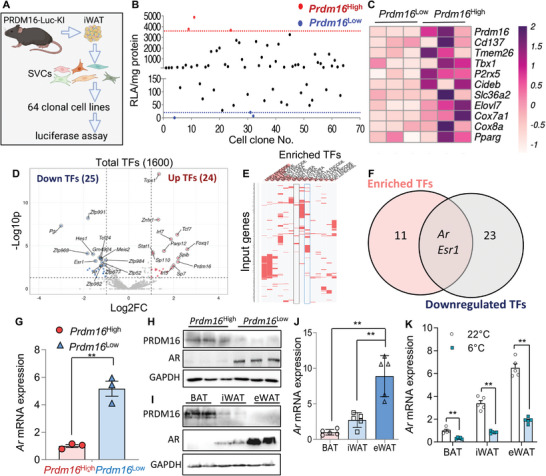
*Prdm16* is negatively correlated with androgen receptor (*Ar*) in adipose tissue. A) Schematic diagram of clonal analysis in SVC from iWAT of PRDM16‐Luc KI mice. B) RLA of 64 clonal cell lines. C–F) *Prdm16^H^* ^igh^ and *Prdm16^L^* ^ow^ cells were subjected to RNA‐Seq. C) Heatmap showing beige‐related genes. D) Volcano plot of all the transcription factors (TF) (*Prdm16^H^* ^igh^ versus *Prdm16^L^* ^ow^). E) ENCODE and ChEA TF enrichment analysis in significantly downregulated genes. F) Venn diagram of downregulated TFs and enriched consensus TFs in (E). G) mRNA levels of *Ar* in SVC clones (*n* = 3/group). H,I) Protein expression of PRDM16 and AR in SVC clones (*n* = 3/group) (H) and in adipose tissues (I). J) mRNA expression of *Ar* in adipose tissues (*n* = 5/group). K) *Ar* mRNA expression in mouse adipose tissues housed at ambient or cold temperature (*n* = 5/group). Data are presented as mean ± SEM; statistical significances between groups were assessed by two‐sided unpaired Student's *t*‐test (G), one‐way ANOVA (J), and two‐way ANOVA (K); *p < 0.05, **p < 0.01.

To better understand the regulatory mechanism underpinning PRDM16 transcription in WAT, we used PRDM16‐Luc KI reporter mice to quantitate PRDM16 expression at single cell resolution. To this end, SVCs in iWAT from PRDM16‐Luc KI mice were isolated, immortalized, followed by limiting dilution, and eventually 64 mono‐clones were obtained (**Figure** **2**A). The cell clones displayed a wide range of luminescence (log2) (Figure 2B), suggesting high heterogeneity in *Prdm16* expression among iWAT SVCs.

To explore the mechanism underlying the differential regulation of PRDM16, clones with the top three highest (*Prdm16* ^High^) and lowest (*Prdm16* ^Low^) luciferase activities were selected for further analysis (Figure S2A, Supporting Information). RNA‐sequencing showed *Prdm16* ^High^ and *Prdm16* ^Low^ cells exhibited distinctive transcription profiles (Figure S2B,C, Supporting Information). In addition to *Prdm16*, a number of other beige cell‐related genes were expressed at substantially higher levels in *Prdm16* ^High^ cells compared to the *Prdm16* ^Low^ cells (Figure 2C). To look for the upstream regulators of *Prdm16* transcription, the TFs showing statistically significant changes between *Prdm16* ^High^ and *Prdm16* ^Low^ cells were identified (Figure 2D). Meanwhile, all upregulated genes were subjected to consensus TF analysis using ENCyclopedia Of DNA Elements (ENCODE) and ChIP‐X Enrichment Analysis (ChEA) Consensus TFs target datasets^[^ ^11^ ^]^ (Figure S2D and Table S1, Supporting Information). However no overlapping transciption factors were found between the enriched transcription factors and upregulated transciption factors (Figure S2E, Supporting Information). Intriguingly, using the same strategy, within downregulated genes and downregulated transcription factors, *Ar* and estrogen receptor 1 (*Esr1*) were identified (Figure 2E,F and Table S2, Supporting Information). Since differentially expressed genes were much more enriched for targets of AR (50 genes), than ESR1 (12 genes) (Table S2, Supporting Information), AR was selected for the subsequent study.

AR expression was significantly lower in *Prdm16* ^High^ SVC clones than in *Prdm16* ^Low^ clones (Figure 2G,H). Furthermore, the inverse correlation between *Ar* and *Prdm16* was also valid in different adipose depots with WAT displaying the highest and lowest *Ar* and *Prdm16* expression, respectively (Figure 2I,J). In mouse BAT, AR expression was much lower compared to WAT depots (Figure 2I,J). After cold exposure a remarkable downregulation of *Ar* expression was also observed in all the adipose depots (Figure 2K). These findings demonstrate that *Ar* and *Prdm16* expressions are negatively associated under physiological conditions across adipose depots in mice.

### 2.3 PRDM16 Expression Is Sex Dependent in Human and Mouse Adipose Tissue

**Figure 3:**
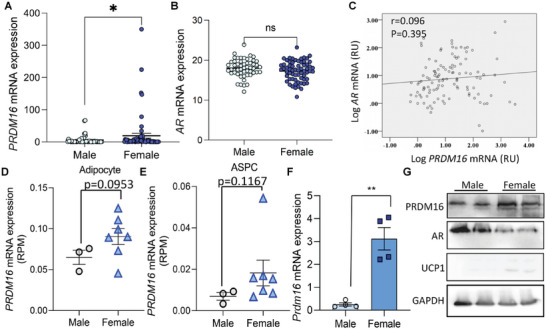
*Prdm16* expression is sex dimorphic in human and mouse adipose tissue. A,B) Relative *PRDM16* (A) and *AR* (B) mRNA expression in human omental adipose tissue. (male *n* = 52, female *n* = 64). C) Pearson correlation plot of *AR* and *PRDM16* mRNA in human omental adipose tissue (*n* = 116). D,E) *PRDM16* mRNA expression in mature adipocytes and adipose tissue precursor cells (ASPC) were extracted from single nuclei sequencing (sn‐Seq) data from human visceral white adipose tissue. D) *PRDM16* mRNA expression in adipocytes of human visceral adipose tissue of both sexes (male *n* = 3, female *n* = 7). E) *PRDM16* mRNA expression in ASPCs of human visceral adipose tissue of both sexes (male *n* = 3, female *n* = 7). F) mRNA expression of *Prdm16* in iWAT from male and female mice (*n* = 4/group). G) Representative western blot result of PRDM16 and AR in mouse iWAT of different sexes. Data are presented as mean ± SEM; statistical significances between groups were assessed by two‐sided unpaired Student's *t*‐test; *p < 0.05, **p < 0.01. ns, not significant. RPM, reads per million mapped reads.

We measured *PRDM16* and *AR* expression in human omental adipose tissue (Table S3, Supporting Information), because this fat depot was reported to possess browning capacity.^[^ ^12^ ^]^ *PRDM16* mRNA expression was highly variable in human omental adipose tissue as compared to *AR* mRNA expression (**Figure** **3**A,B). *PRDM16* expression was significantly higher in the omental adipose tissue of female persons as compared to the male counterparts (Figure 3A). Nevertheless, different from the observations in mice, we did not detect a correlation between the expressions of *AR* and *PRDM16*, nor a sex difference in *AR* expression in human (Figure 3B,C and Figure S3, Supporting Information). Using a recently published single nuclei RNA sequencing (sn‐Seq) dataset of human visceral adipose tissue,^[^ ^13^ ^]^ we checked *PRDM16* expression in adipocyte and adipose tissue precursor cells (ASPC) respectively. In both cell types, a trend of increase of *PRDM16* in female subjects was found (Figure 3D,E). The sex dimorphic expression of *Prdm16* was also evident in mouse iWAT where female mice displayed a higher level of *Prdm16* mRNA and protein, compared to that in male mice (Figure 3F,G). However, *Prdm16* expression in BAT was higher in male mice (Figure S4, Supporting Information), suggesting that its expression is regulated by distinct mechanisms in brown and white adipose tissues.

### 2.4 AR Is a Negative Regulator of *Prdm16* Transcription

**Figure 4:**
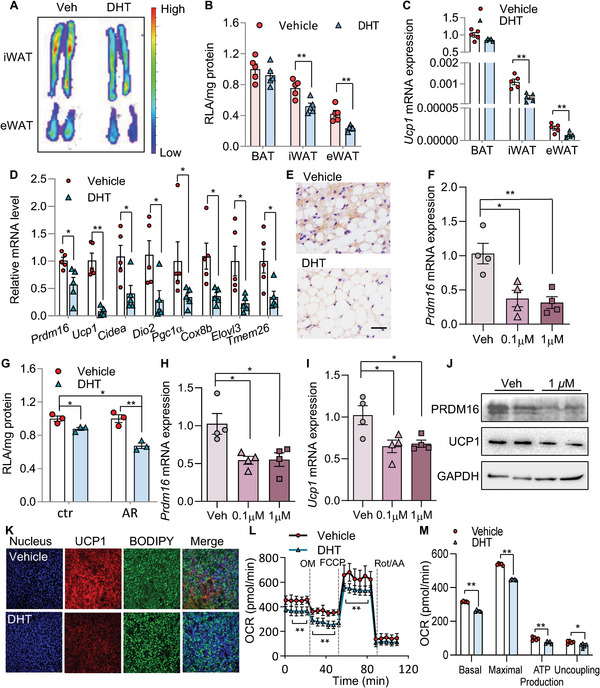
PRDM16 expression and white adipocyte beiging are inhibited by androgen receptor. A–C) PRDM16‐Luc KI mice were treated with DHT or vehicle for 7 days. A) Luminescence images and B) relative luminescence in mouse adipose tissues (B: *n* = 5/group). C) qPCR of *Ucp1* mRNA expression in adipose tissues (*n* = 5/group). D,E) Male C57BL/6J mice were treatment with DHT or vehicle and housed at 6 °C for 7 days. D) qPCR of beige markers and E) immunohistochemical staining of PRDM16 in iWAT. Scale bar: 100 µm. F) *Prdm16* mRNA expression in iWAT SVCs from *Prdm16*‐Luc KI mice treated with DHT for 24 h (*n* = 4/group). G) iWAT SVCs from PRDM16‐Luc KI mice with doxycycline (Dox)‐inducible *Ar* expression were established. The cells with Dox (AR) or without Dox (ctr) were treated with DHT (1 µm) or vehicle for 24 h before luminescence was determined (*n* = 3/group). H–M) iWAT SVCs from wild‐type mice were differentiated to beige adipocytes and treated with DHT for 24 h. Relative mRNA levels of *Prdm1*6 (H) and *Ucp1* (I) in adipocytes (*n* = 4/group). J) PRDM16 and UCP1 protein expression in adipocytes. K) Immunofluorescence staining of UCP1 in beige adipocytes. L,M) Oxygen consumption rate (OCR) was measured by seahorse bioanalyzer (*n* = 5/group). Data are presented as the mean ± SEM; statistical significances between groups were assessed by two‐sided unpaired Student's *t*‐test (D), one‐way ANOVA (F,H,I), and two‐way ANOVA (B,C,G,L,M);*p < 0.05, **p < 0.01.

To investigate whether AR is an upstream regulator of PRDM16, male PRDM16‐Luc KI mice were administered with dihydrotestosterone (DHT, a potent and non‐aromatizable androgen, 10 mg kg^−1^ d^−1^, 7 days). Following this treatment regime luciferase activity in WATs (including iWAT and eWAT), but not in BAT, was suppressed as compared to those receiving the vehicle solution (**Figure** **4**A,B). Consistent with this finding, expression of *Ucp1* was significantly downregulated in iWAT and eWAT after DHT treatment (Figure 4C). Likewise, when wild‐type (WT) C57BL/6J mice were orally supplemented with DHT and housed at cold temperature, mRNA expressions of *Ucp1* and other beige cell related genes were uniformly decreased, accompanied by mitigated biogenesis of multilocular adipocytes and reduced expression of PRDM16 (Figure 4D,E and Figure S5A, Supporting Information). These findings indicate that promoting AR signaling inhibits *Prdm16* transcription and WAT beiging in vivo.

In in vitro cultured SVCs, DHT treatment suppressed *Prdm16* mRNA expression level by ≈60% (Figure 4F). Similarly in SVCs from PRDM16‐Luc KI mice, DHT suppressed *Prdm16*‐driven luciferase activity (Figure 4G). More importantly, luminescence was further decreased upon doxycycline (Dox)‐induced AR overexpression (Figure 4G). Treatment of DHT in beige adipocytes also led to decreased *Prdm16* and *Ucp1* expressions (Figure 4H–K), and this effect was completely abolished in *Ar* knockout (KO) beige adipocytes (Figure S5B, Supporting Information). These gain‐of‐function and loss‐of‐function studies show that the suppressive effect of DHT is AR‐dependent.

Functionally, beige adipocytes displayed decreased basal and maximal oxygen consumption rate (OCR) after DHT treatment, which was attributed to both coupled and uncoupled respiration (Figure 4L,M). This is in line with previous reports that PRDM16 contributes to both UCP1‐dependent and UCP1‐independent energy consumption.^[^ ^8^, ^14^ ^]^ Collectively these results corroborate the notion that AR activation plays an essential role in regulating *Prdm16* expression in ASPCs and mature adipocytes in WAT.

### 2.5 AR Directly Binds at the *Prdm16* Locus and Inhibits WAT Browning via Suppression of PRDM16

**Figure 5:**
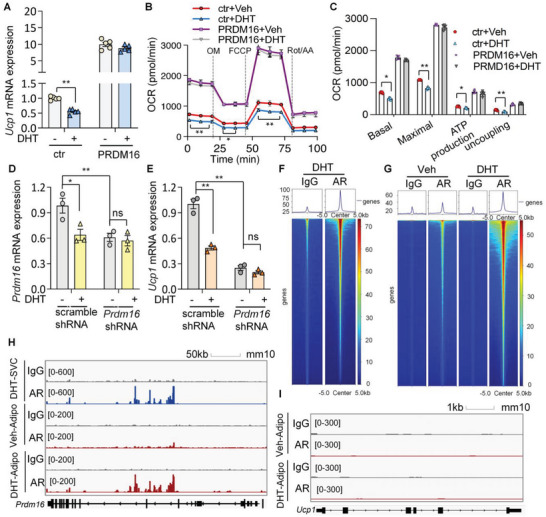
Androgen inhibits white adipocyte beiging via suppression of *Prdm16* expression. A–C) iWAT SVCs were transfected with a Dox‐inducible *Prdm16* cassette (PRDM16) and differentiated to beige adipocytes, followed by treatment with DHT (1 µm) or vehicle for 24 h. A) mRNA expression of *Ucp1* (*n* = 5/group). B,C) OCR in adipocytes (*n* = 5/group). D,E) iWAT SVCs expressing scrambled shRNA or shRNA targeting *Prdm16* were differentiated to beige adipocytes and treated with DHT (1 µm) or vehicle for 24 h. mRNA expression of D) *Prdm16* and E) *Ucp1* (*n* = 3/group). F–I) SVCs and mature beige adipocytes were subjected to CUT&Tag analysis for AR. F,G) Heat map illustrating the signal intensity of AR‐binding. The *x*‐axis represents read densities within 5‐kb region around the peak summit; the *y*‐axis represents each predicted binding site. H,I) Genome browser tracks showing enrichment of reads in *Prdm16* (H) and *Ucp1* loci (I). Data are presented as the mean ± SEM; statistical significances between groups were assessed by two‐way ANOVA; *p < 0.05, **p < 0.01.

Despite the suppression of UCP1 by DHT in beige adipocytes, this effect was abolished upon overexpression of PRDM16 (**Figure** **5**A). Likewise, PRDM16 overexpression reversed the DHT‐evoked downregulation of OCR in beige adipocytes (Figure 5B,C). Furthermore, when PRDM16 was knocked down in beige adipocytes, the suppressive effects of DHT on *Prdm16* and *Ucp1* expression were largely abolished (Figure 5D,E). Taken together these data showed that AR mitigates white adipocyte beiging through its suppressive action on PRDM16. Cleavage under targets and tagmentation (CUT&Tag) is an antibody‐tethered tagmentation strategy that maps transcription‐coupled accessible sites at high resolution.^[^ ^15^ ^]^ Using this method, we mapped the genome‐wide AR binding sites in SVCs and mature adipocytes (Figure 5F–I and Figure S6, Supporting Information). Upon agonism of AR by DHT, the peak intensity of AR binding sites was significantly substantiated in beige adipocytes (Figure 5G). More importantly, AR occupancy was readily observed in a number of genomic regions within the intron of *Prdm16* locus (Figure 5H). In contrast, no AR binding was detected at *Ucp1* and a number of other beige marker gene loci (Figure 5I and Figure S7, Supporting Information), suggesting that AR directly modulates *Prdm16*, but not *Ucp1* transcription, via genomic actions.

### 2.6 Mice with Adipocyte‐Selective Deletion of *Ar* Are More Susceptible to WAT Beiging while Overexpression of AR in Adipocytes Mitigates Beiging

**Figure 6:**
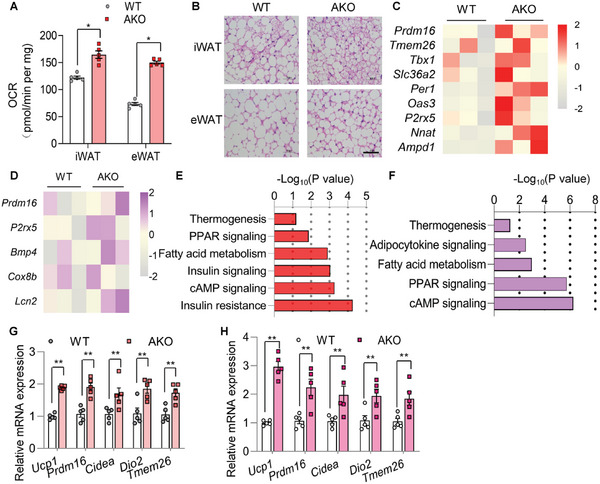
Adipocyte‐selective *Ar* gene knockout enhances WAT beiging in mice. 8‐week‐old male WT and AKO mice were subjected to cold exposure for 3 days. A) OCR in WAT of WT and AKO mice (*n* = 5/group). B) Representative images of HE staining in iWAT and eWAT. Scale bar: 50 µm. C–F) RNAseq analysis of iWAT and eWAT of WT and AKO mice. C,D) KEGG pathway analysis of the RNAseq data of iWAT (C) and eWAT (D). E,F) Heatmap of beiging genes in iWAT (E) and eWAT (F). G,H) qPCR analysis of the thermogenic genes in iWAT (G) and eWAT (H) (*n* = 5/group). Data are presented as the mean ± SEM; statistical significances between groups were assessed by two‐way ANOVA (A) and two‐sided unpaired Student's *t*‐test (G,H); *p < 0.05, **p < 0.01.

To examine the regulatory role of AR on PRDM16 in adipocytes in vivo as well as its physiological consequence, adipocyte‐selective *Ar* KO mice by mating *Ar* flox/flox mice with *AdipoQ*‐Cre transgenic mice (termed AKO) were used in our study (Figure S8A,B, Supporting Information). Both AKO and WT mice showed similar food intake and body weight (Figure S8C,D, Supporting Information). However, when mice were challenged with cold temperature, iWAT and eWAT from AKO mice exhibited a higher respiration rate (**Figure** **6**A), as well as a higher percentage of multilocular cells induced in iWAT (Figure 6B). Notably, in eWAT which is normally resistant to cold‐induced beiging, multilocular adipocytes were observed in AKO mice (Figure 6B).

We used RNA‐Seq to profile the WAT transcriptomic landscape. These analyses demonstrated elevations in a panel of beiging and thermogenesis‐related genes in iWAT and eWAT from AKO mice, including *Prdm16* (Figure 6C,D). KEGG pathway enrichment revealed that thermogenesis, PPAR signaling, fatty acid metabolism, and cAMP signaling pathways were among the top processes upregulated in iWAT and eWAT of AKO mice (Figure 6E,F). The elevation of *Prdm16* and other beiging related genes in WAT of AKO mice was validated by qPCR (Figure 6G,H). In contrast, the expression of *Prdm16* and other browning related genes in BAT was comparable between WT and AKO mice (Figure S8E, Supporting Information), suggesting that AR primarily regulates PRDM16 and adipose browning in WAT, but not in BAT in vivo.

**Figure 7:**
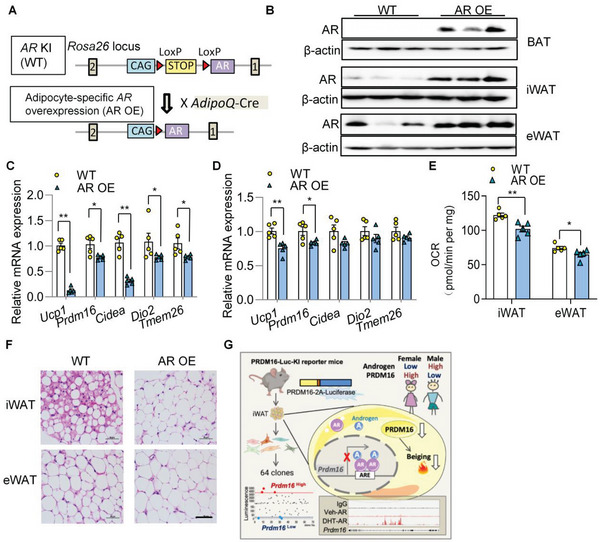
Adipocyte‐selective overexpression of AR blunts WAT beiging in mice. A) Schematic diagram of AR OE mice generation. A CAG promoter‐LoxP‐stop codon‐LoxP‐human *AR* orf cassette was knocked into the *Rosa26* locus. The mice were mated with AdipoQ‐Cre transgenic mice to obtain the adipocyte selective *AR* overexpressing mice (AR OE). The *AR* KI mice without Cre were used as the WT control. B) Adipose depots from 8‐weeks‐old male AR OE and WT mice were isolated for validation of *AR* expression. Western blotting of AR expression in adipose depots of the WT and AR OE mice. C–F) 8‐week‐old male WT and AR OE mice were housed at cold temperature for 3 days. C,D) qPCR analysis of the thermogenic genes in iWAT (C) and eWAT (D) (*n* = 5/group). E) OCR in WAT (*n* = 5/group). F) Representative images of HE staining of iWAT and eWAT. Scale bar: 50 µm. Data are presented as the mean ± SEM; statistical significances between groups were assessed by two‐sided unpaired Student's *t*‐test (C,D) and two‐way ANOVA (E); *p < 0.05, **p < 0.01. G) Hypothetical diagram of the study.

To further leverage the physiological function of AR using gain‐of‐function approach, we generated adipocyte‐selective *AR* overexpressing mice (**Figure** **7**A). Overexpression of AR in adipose depots was verified by qPCR and western blotting (Figure 7B and Figure S9A, Supporting Information). When challenged with cold temperature, AR OE mice exhibited significantly lower expression level of *Prdm16* in iWAT and eWAT, along with other browning markers (Figure 7C,D). In contrast, overexpression of AR did not alter *Prdm16* expression in BAT (Figure S9B, Supporting Information). This finding again implied that AR regulates PRDM16 expression in a WAT‐selective manner while in BAT the transcription of *Prdm16* is regulated by other mechanisms. OCR was suppressed in iWAT and eWAT of AR OE mice (Figure 6E), consistent with the observation that the number of multilocular beige adipocytes was lower in iWAT of the AR OE mice (Figure 7F). AR OE mice also exhibited an enlarged adipocyte size in eWAT (Figure 7F). Taken together, results from both adipocyte‐specific gene deletion and overexpression mouse models unambiguously demonstrated that AR is a negative regulator of *Prdm16* transcription in WAT (Figure 7G).

## 3 Discussion

PRDM16 plays a key role in brown remodeling in both BAT and WAT.^[^ ^5^, ^6^, ^7^, ^8^ ^]^ Furthermore, PRDM16 is implicated in UCP1 and adipose browning‐independent processes, such as adipose fibrogenesis and sympathetic innervation.^[^ ^10^ ^]^ Clinical studies have also shown that the amount of PRDM16 is associated with obesity and diabetes. Collectively PRDM16 is a viable target to treat obesity and diabetes. However, in contrast to the understanding on its physiological functions, much less is known as to how PRDM16 expression is regulated. Muscle enriched miR‐133 directly targets the 3′UTR of *Prdm16* and negatively regulates PRDM16 expression, which in turn modulates the fate choice between myogenic and brown adipose precursors, as well as promotes differentiation of ASPC to mature brown adipocytes.^[^ ^16^ ^]^ At the post‐translational level, PRDM16 is sumoylated such that its ubiquitination‐mediated degradation is blocked.^[^ ^17^ ^]^ Recently, Wang et al. identified CUL2–APPBP2 as the ubiquitin E3 ligase that determines PRDM16 protein stability by catalyzing its ubiquitination.^[^ ^18^ ^]^ Early B cell factor‐2 (EBF2) was identified as a brown adipose‐enriched factor and recruits PPAR*γ* to all of its brown‐adipose selective binding sites including *Prdm16* and *Ucp1* and thus maintains the brown‐specific characteristics.^[^ ^19^ ^]^ Using clonal analysis, we found AR as the most potent hit within the negative regulators of PRDM16 transcription. Notably, compared to PPAR*γ*/EBF2, which are the more general modulators of adipose browning, the negative regulation directly elicited by AR is highly selective since a number of other brown and beige‐genes including *Ucp1*, *Dio2*, *Tnfrsf9*, and *Tmem26* are not the direct target of AR, as evidenced by our CUT&Tag analysis. Recently a study reported the general inhibitory effect of androgens on WAT thermogenic capacity and the downregulation of browning marker genes including *Prdm16*, *Ucp1*, and *Dio2*.^[^ ^20^ ^]^ Considering the role of PRDM16 at the apex of WAT brown remodeling, the suppressive effect of androgens on WAT browning is likely secondary to the direct transcriptional blockade of PRDM16. Indeed, our study demonstrated that overexpression of PRDM16 overrides the inhibition of *Ucp1* expression and suppression of oxygen consumption induced by DHT in beige adipocytes. Therefore to our knowledge this is the first study to uncover the selective and negative regulation of PRDM16 at the transcriptional level.

Androgens play an important role in regulation of body fat distribution and function. Androgen excess, more commonly seen in female (such as polycystic ovary syndrome, PCOS) and prepubertal children, is significantly correlated with body mass index, abdominal obesity, insulin resistance, metabolic derangements, and subclinical cardiovascular disease.^[^ ^21^ ^]^ Hyperandrogenemia predicts a worse metabolic outcome in PCOS patients.^[^ ^22^ ^]^ Amelioration of hyperandrogenism, either using antiandrogen or oral contraceptive pill, improves abdominal adiposity and metabolic disorders.^[^ ^23^ ^]^ Androgen administration in obese postmenopausal women or female rhesus macaques causes a significant gain in visceral fat and accelerates adipose tissue dysfunction.^[^ ^24^ ^]^ Although, in males obesity seems more commonly associated with hypogonadism, this is likely because that in males testosterone is aromatized into 17*β*‐estradiol (E2) for energy homeostasis. Indeed, while orchidectomized male rodents treated with either testosterone or E2 remain lean, those treated with the non‐aromatizable androgen DHT develop obesity and glucose intolerance.^[^ ^25^ ^]^ In the same vein, a large scale interventional study on male subjects reported that the percentage of body fat increases in the group receiving testosterone daily together with aromatase inhibitor (to inhibit the aromatization of testosterone to estradiol),^[^ ^26^ ^]^ indicating that activation of AR favors body fat accumulation while much of the beneficial effects observed in androgen therapy in male is mediated by aromatized estradiol. Despite these clinical observations, the mode of action of androgen in WAT is still obscure.

Our work offers a new foundation for understanding the physiological function and working mechanism of androgens and AR in WAT. Sex differences in beige cell activity has been observed in animal models.^[^ ^27^ ^]^ In adult human, whether beige adipocytes in human is sex‐dependent awaits future investigations, but fluorodeoxyglucose‐PET/CT scan‐based studies, which cannot differentiate between beige and brown adipocytes, reveal sex as an independent determinant of BAT activity with women having more often detectable BAT.^[^ ^28^ ^]^ Different from mice, in human omental WAT possesses the browning capacity,^[^ ^12^ ^]^ coincided with the recent sn‐Seq result that beige‐like adipocytes were detected in visceral WAT but not in subcutaneous WAT in human.^[^ ^13^ ^]^ Our study uncovers a higher level of *PRDM16* in omental WAT of female individuals and this thus intriguingly raises the possibility that this gender difference on PRDM16 partially explains the higher incidence of visceral obesity in males and post‐menopausal females. Furthermore, considering the involvement of PRDM16 beyond controlling UCP1 and beiging, such as adipose fibrogenesis and sympathetic innervation,^[^ ^10^ ^]^ further studies will have to determine whether the androgen/AR‐mediated suppression of PRDM16 underlies those biological processes in WAT.

Our results showed in both humans and mice, PRDM16 exhibit sex dimorphism, demonstrating that AR activity is negatively associated with PRDM16 expression in both species. Furthermore in mice the expressions of *Ar* and *Prdm16* were also negatively correlated, but not in human. The exact mechanism for such discrepancy is unclear. It is likely attributed to the fact that in human *AR* mRNA expression is comparable between two sexes while in mice *Ar* is expressed at a higher level in male than in female. This suggests the mechanism regulating the mRNA expression of AR is different in human and mouse which awaits future investigation.

Cre‐loxP‐mediated deletion of the *Ar* gene using *aP2*‐Cre has been reported to exhibit normal weight and adiposity,^[^ ^29^ ^]^ but this mice strain also displays a partial deletion of *Ar* in the brain,^[^ ^29a^ ^]^ which confounded the study. In the current study, a more stringent adipocyte‐selective *Ar* KO mice carrying *AdipoQ* promoter‐driven Cre recombinase were used. Together with adipocyte‐selective AR over‐expressing mice, we were at a better position to interrogate the function of androgen and AR in adipose tissue. It is interesting to find that the regulation of androgen on PRDM16 is depot specific. Ablation of AR signaling augments PRDM16 expression and beige biogenesis in iWAT and even eWAT. In contrast, the suppressive action of AR on PRDM16 is invalid in BAT, since neither supplementation of AR agonist, adipocyte‐selective deletion nor overexpression of AR, alters PRDM16 expression in mouse BAT. The unresponsiveness of brown adipocytes to androgen in PRDM16 suppression seems to be intrinsic and is downstream of AR since overexpression of AR in BAT still failed to downregulate PRDM16 expression in mice. The mechanism is currently unclear. PRDM16 expression is much higher in BAT compared to WAT, suggesting that the mechanisms regulating PRDM16 expression in these fat depots are distinct. In BAT, the action of those positive regulators of PRDM16 dominates, which is too strong to be overridden by AR, considering that the expression of AR is low in BAT. Nonetheless, it is still worthwhile to map the binding sites of AR on PRDM16 in BAT in future, which will offer additional insights on the actions of androgens in BAT.

In sum, because of the global rise in overweight, obesity, and metabolic syndromes, it is imperative to improve our understanding on the energy utilization in WAT, especially visceral WAT. The current revelation of androgen in negative modulation of PRDM16 would potentially provide a good therapeutic window for anti‐obese and anti‐metabolic disease drugs targeting PRDM16.

## 4 Experimental Section

### Mice

PRDM16‐Luc KI mice (KI) were generated by deleting the stop codon of mouse *Prdm16* gene and replacing it with T2A peptide and firefly luciferase. *Ar* flox/flox mice were generated by floxing exon 2 of mouse *Ar* gene through homologous recombination. Transgenic mice with Cre recombinase driven by the *adipoQ* promoter (AdipoQ‐Cre transgenic mice) were crossed with the *Ar* flox/flox mice to generate adipocyte‐selective knockout of *Ar* mice (AKO), which selectively abolished *Ar* gene expression within adipocytes. CRISPR/Cas9 technology was used to insert a CAG‐LoxP‐STOP‐LoxP‐AR expression cassette at the Rosa26 gene locus of wild‐type C57BL/6J mice (*AR* KI mice). *AdipoQ*‐Cre transgenic mice were mated with *AR* KI mice in which the expression of human *AR* sequence was turned on by Cre‐mediated deletion of a STOP cassette between the CAG promoter and human *AR* coding sequence (AR OE). All above‐mentioned transgenic mice were on a C57BL/6J background. All animal experiments were conducted in accordance with the Guide for the Care and Use of Laboratory Animals and approved by the Animal Care and Use Committee of Guangzhou Institutes of Biomedicine and Health, Chinese Academy of Science, and Animal Experimentation Ethics Committee of The Chinese University of Hong Kong (21‐051‐MIS). Mice were housed in a specific‐pathogen‐free environment (12 h light/dark cycle, 22 ± 1 °C, 60–70% humidity) with free access to food and water. 8‐week‐old male mice were used for experiments unless specified otherwise. For cold exposure, mice were housed at 6 °C, and those housed at 22 °C were used as the control. For DHT supplementation, mice were orally given equal volume of DHT (10 mg kg^−1^ d^−1^) or vehicle (oil) for 7 days.

### Human Subjects

The study enrolled 116 patients (male *n* = 52, female *n* = 64) undergoing laparoscopic cholecystectomy for gallstone and gallbladder polyps between 2019 and 2021 at the research institute of general surgery from Jinling Hospital. Participants were excluded if they had 1) acute infection or systemic inflammatory disease or 2) severe organ dysfunction of liver, kidney, and heart. The study protocol was approved by the Ethics Committee of the Jinling Hospital (2019NZKY‐008‐03) and all participants were provided with the informed written consent prior to the procedure. The omental adipose tissue was dissected and flash frozen in liquid nitrogen.

### Luciferase Imaging and Luciferase Activity Analysis

Mice were intraperitoneally injected with D‐luciferin (150 mg kg^−1^, Promega). The mice and the adipose tissues were harvested 15 min later and the bioluminescence imaging was performed with an IVIS Imaging Device (Xenogen Corp.). The results were quantified by Living Image software (PerkinElmer). Luciferase activity of cells and tissues was measured using the Steady‐Glo Luciferase Assay (Promega) according to the manufacturer's instructions. The signal was measured with EnSpire Alpha 2390 (PerkinElmer) and normalized with protein concentrations.

### Isolation of SVC and Construction of Clonal Cell Lines

SVCs were obtained by dissecting iWAT from PRDM16‐Luc KI mice. The dissected iWAT was rinsed in PBS, minced, and digested for 40 min at 37 °C in 0.1% w/v Collagenase Type I (Sigma‐Aldrich) with D‐Hanks buffer. Digested tissues were filtered through a 250 µm nylon mesh and centrifuged at 800 × *g* for 3 min. The pellet was resuspended in Dulbecco's modified Eagle's medium (high glucose, with L‐glutamine, Gibco) with 10% fetal bovine serum (HyClone). Cells were immortalized by infecting the retrovirus expressing SV40 large T antigen gene. Limiting dilution method was used to establish 64 clonal cell lines.

### RNA Extraction and Realtime PCR

Total RNA was extracted with Trelief RNAprep FastPure Tissue&Cell Kit (Tsingke). First‐strand cDNA was synthesized using the TransScript Uni All‐in‐One First‐Strand cDNA Synthesis SuperMix for qPCR (TransGen Biotech) with 1 µg of RNA as the template for each reaction. mRNA levels were quantified under optimized conditions with Hieff qPCR SYBR Green Master Mix (Yeasen) according to the manufacturer's instructions. *Rps18* and *RPS18* were used as the reference genes for mouse and human samples, respectively.

### Western Blotting

Cells and adipose tissues were lysed in RIPA Lysis (Servicebio) containing PMSF (Beyotime). Lysates were resolved by 10% SDS‐PAGE and transferred onto a PVDF membrane, blotted with antibodies to PRDM16 (Abcam), AR (Abcam), UCP1 (Abcam), luciferase (Abcam), *β*‐actin (Cell Signaling Technology), and GAPDH (Cell Signaling Technology). HRP‐linked anti‐rabbit IgG (Cell Signaling Technology) was used as the secondary antibody. The signals were developed using SuperSignal West Femto Maximum Sensitivity Substrate (Thermo Fisher Scientific).

### Seahorse Analysis

The OCR of cultured adipocytes and adipose tissues was measured using Seahorse XF Cell Mito Stress Test Kit (Agilent) and analyzed by the XFe24 Seahorse bioanalyzer (Agilent). Adipocytes were treated with DHT (Meilunbio) or DMSO for 24 h and then equilibrated in carbonate free medium and incubator for 1 h. After measuring basal levels of OCR, the following drugs were sequentially loaded to each well: oligomycin (5 µm), FCCP (5 µm), rotenone (3 µm), antimycin (5 µm). 2 mg of adipose tissues were equilibrated at 37 °C in carbonate free condition for 1 h before measurement.

### Generation of Stable Transfectants with Inducible Gene Expression

To generate *Prdm16* and *Ar* stable transfectants in PRDM16‐Luc SVCs, plasmids (PB‐TRE‐*Prdm16* and PB‐TRE‐Flag‐*Ar*) were transfected into SVCs via electroporation using NuclofZctor II (A023, Lonza). The cells were incubated for 24 h and screened in selection media containing puromycin (1.2 µg mL^−1^, Sigma‐Aldrich) for 3 days. Dox (1.5 µg mL^−1^) was used for inducing PRDM16 and AR overexpression.

### *Prdm16* Knocking Down

shRNA‐mediated *Prdm16* knocking down was performed following a previous publication^[^ ^5^ ^]^ and the construct was purchased from Addgene (#15505). The siRNA sequences used were as follows: scramble shRNA: 5′‐GCGGAGAAAGUGGAUUUAU‐3, *Prdm16*‐shRNA: 5′‐GAAGAGCGUGAGUACAAAU‐3′. After electroporation into the immortalized adipose SVCs, the cells were selected for positive transfectants with G418 for 5 days. The G418‐resistant SVCs were differentiated to beige adipocytes in vitro.

### RNA‐Seq

RNA‐seq was performed by Shanghai Majorbio Bio‐pharm Technology Co., Ltd. using Illumina HiSeq X10 (Illumina). Significance analysis (two fold change and *p*‐value < 0.05) of results was used to identify genes strongly up‐ or down‐regulated by CINPs using unlogged data and with a false discovery rate < 0.05. Fold‐change was calculated with the average transcript levels compared to control values that were in turn log2‐transformed and calculated for Spearman correlation coefficients between treatments. All query sequences were queried against commonly used databases by BLASTx search to identify homologues.

### CUT&Tag

CUT&Tag analysis was performed with Hyperactive Universal CUT&Tag Assay Kit for Illumina (Vazyme) following the manufacturer's instructions. Cells were harvested, counted (10 000 cells), and centrifuged for 3 min at 600 × *g* at room temperature. Cell nucleus were prepared with NE buffer and resuspended in Wash buffer. Activated ConA Beads were added to samples and incubated at room temperature for 10 min. AR antibody (Cell Signaling Technology) and control IgG (Cell Signaling Technology, negative control) were incubated with the samples on a rotating platform overnight at 4 °C. Goat anti‐rabbit IgG H&L (Abcam) diluted in Dig‐Wash buffer was incubated with the sample at room temperature for 1 h. After washing with Dig‐Wash buffer, pA‐Tn5 adapter complex was added with gentle vortexing and incubated at room temperature for 1 h, and washed with Wash buffer. The samples were resuspended in Tagmentation buffer, incubated at 37 °C for 1 h and STOP buffer was added and incubated at 55 °C for 30 min and 70 °C for 20 min. DNA was extracted according to the protocols. The library DNA was purified and amplified for sequencing with the Illumina NovaSeq 6000. To amplify libraries, 15 µL of DNA was mixed with 25 µL of 2× CAM and 5 µL of a universal i5 and a uniquely barcoded i7 primer, using a different barcode for each sample. The sample was placed in a Thermocycler (Bio‐Rad) with a heated lid using the following cycling conditions: 72 °C for 3 min; 95 °C for 3 min; 14 cycles of 98 °C for 10 s and 60 °C for 5 s; final extension at 72 °C for 1 min and hold at 4 °C. Post‐PCR clean‐up was performed by adding VAHTS DNA Clean Beads.

### Hematoxylin and Eosin Staining, Immunohistochemistry, and Immunofluorescence Staining

Adipose tissues were fixed in 4% formaldehyde overnight. After paraffin embedding and sectioning at 5 µm, the sections were stained with hematoxylin and eosin (Beyotime) according to standard protocols. For immunohistochemistry, the sections were blocked with 5% BSA in PBS for 1 h, and incubated with anti‐PRDM16 antibody (R&D systems) at 4 °C overnight. The results were imaged with a light microscopy Motic BA600 (Motic Germany GmbH) and a slice scanner Pannoramic MIDI II (3DHISTECH). PRDM16‐Luc SVCs were differentiated into adipocytes and treated with DHT (1 µm, Meilunbio) or DMSO for 24 h. The adipocytes were washed with PBS, fixed in 4% formaldehyde for 10 min, permeabilized in PBST (PBS +0.1% TritonX‐100) for 10 min, blocked in 5% BSA for 30 min on ice, and incubated with Anti‐UCP1 antibody (Abcam) at 4 °C overnight. The 2nd day, after washing for three times, the adipocytes were incubated with fluorescently labeled goat anti‐rabbit IgG (Thermo Fisher Scientific) for 1 h, followed by incubation with BODIPY 493/503 (Thermo Fisher Scientific) plus Hoechst 33342 (Merck) for 20 min. Laser scanning microscopy 800 (ZEISS) with oil‐immersion objective lens was used for confocal imaging.

### Statistical Analysis

The results were presented as means ± standard error of mean (SEM). For animal and cell‐based studies, statistical analysis was performed with GraphPad Prism 7. After calculating normality by D'Agostino–Pearson omnibus test, two‐sided unpaired *t*‐test was used to compare two groups of samples. One‐way or two‐way ANOVA analysis followed by Tukey's HSD post hoc test was used for multiple group comparisons. For analysis of mRNA levels of genes in human sample, SPSS 22.0 software was used for the statistical analyses. Relative mRNA expression of *PRDM16* and *AR* was log transformed to achieve a more normal distribution and Pearson and partial correlation coefficient was used for analysis of relation between two variables. *p* < 0.05 was regarded as significant. Sample size and *p*‐values are described in the figure legends.

## Conflict of Interest

The authors declare no conflict of interest.

## Author Contributions

S.Z., T.N., and L.L. contributed equally to this work. H.X.H., D.W., T.N., and S.Z. designed the study. S.Z., T.N., L.L., P.G., Y.Z., W.S., Z.L., Q.L., Y.Q., W.W., and M.X. performed the experiments. S.Z., T.N., L.L., and P.G. analyzed the data. Q.Lo. performed the bioinformatics analyses. H.X.H., D.W., S.Z., T.N., K.L., and C.C. wrote and edited the manuscript and all authors reviewed and approved the manuscript. H.X.H., D.W., and T.N. supervised the study.

## Supporting information

Supporting Information

Supplemental Table 1

Supplemental Table 2

Supplemental Table 3

## Data Availability

The data that support the findings of this study are available from the corresponding author upon reasonable request.
