# Supplementary material for: Androgen Receptor is a Negative Regulator of PRDM16 in Beige Adipocyte
Source: Adv Sci (Weinh). 2023 May 21;10(21):2300070. doi: 10.1002/advs.202300070 (PMC10375129; doi:10.1002/advs.202300070)
Supplement: Supplementary file 1 — Supporting Information [file ADVS-10-2300070-s003.pdf]

ADVANCED SCIENCE
Open Access

Supporting Information

for *Adv. Sci.*, DOI 10.1002/advs.202300070

Androgen Receptor is a Negative Regulator of PRDM16 in Beige Adipocyte

Shiting Zhao, Tao Nie, Lei Li, Qiaoyun Long, Ping Gu, Yuwei Zhang, Wei Sun, Zexin Lin, Qing Liu, Yue Qi, Wei Wang, Mengyuan Xie, Kerry Loomes, Chenleng Cai, Donghai Wu* and Hannah Xiaoyan Hui**

Supporting Information

Androgen Receptor is a Negative Regulator of PRDM16 in Beige Adipocyte

Shiting Zhao, Tao Nie, Lei Li, Qiaoyun Long, Ping Gu, Yuwei Zhang, Wei Sun, Zexin Lin, Qing Liu, Yue Qi, Wei Wang, Mengyuan Xie, Kerry Loomes, Chenleng Cai, Donghai Wu*, Hannah Xiaoyan Hui**

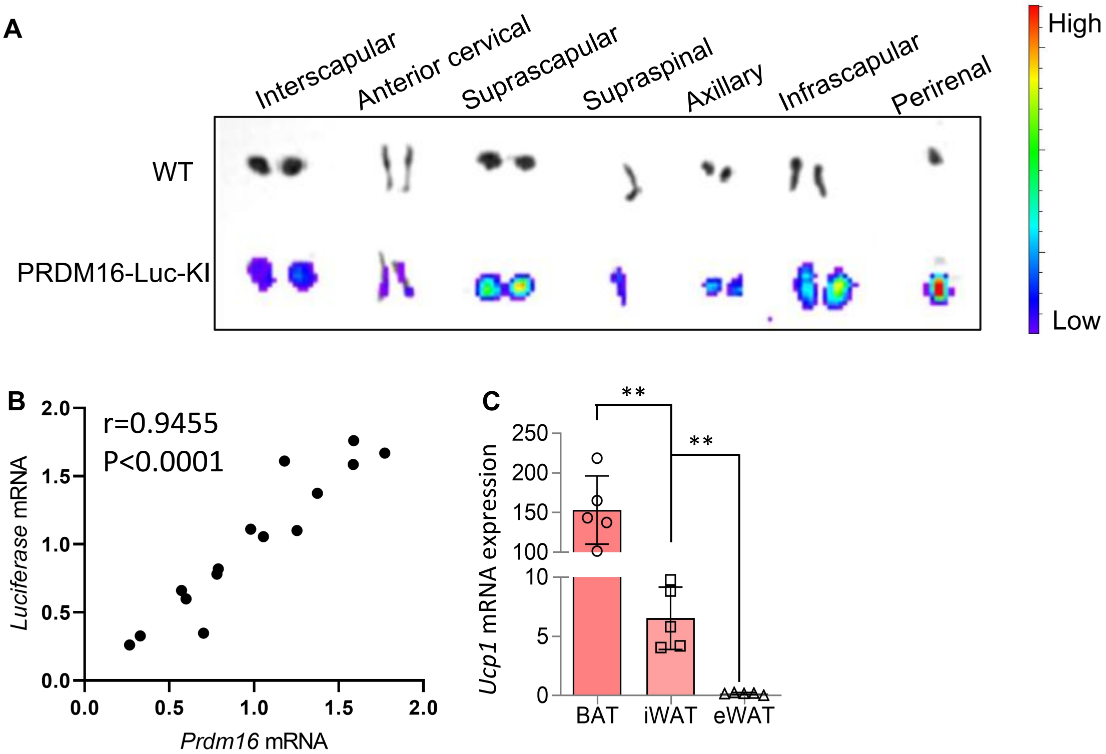

Figure S1. Establishment of a luciferase-based PRDM16 reporter knockin mouse model.
(A) Luminescence imaging of various BAT from WT and KI mice. (B) Correlation of mRNA expressions of *Prdm16* and *Luciferase* in iBAT, iWAT and eWAT of the mice (n=15). (C) qPCR analysis of *Ucp1* mRNA in different adipose tissues (n=5/group). Data are presented as mean ± SEM; statistical significances between groups were assessed by one-way ANOVA; $^{**}P < 0.01$.

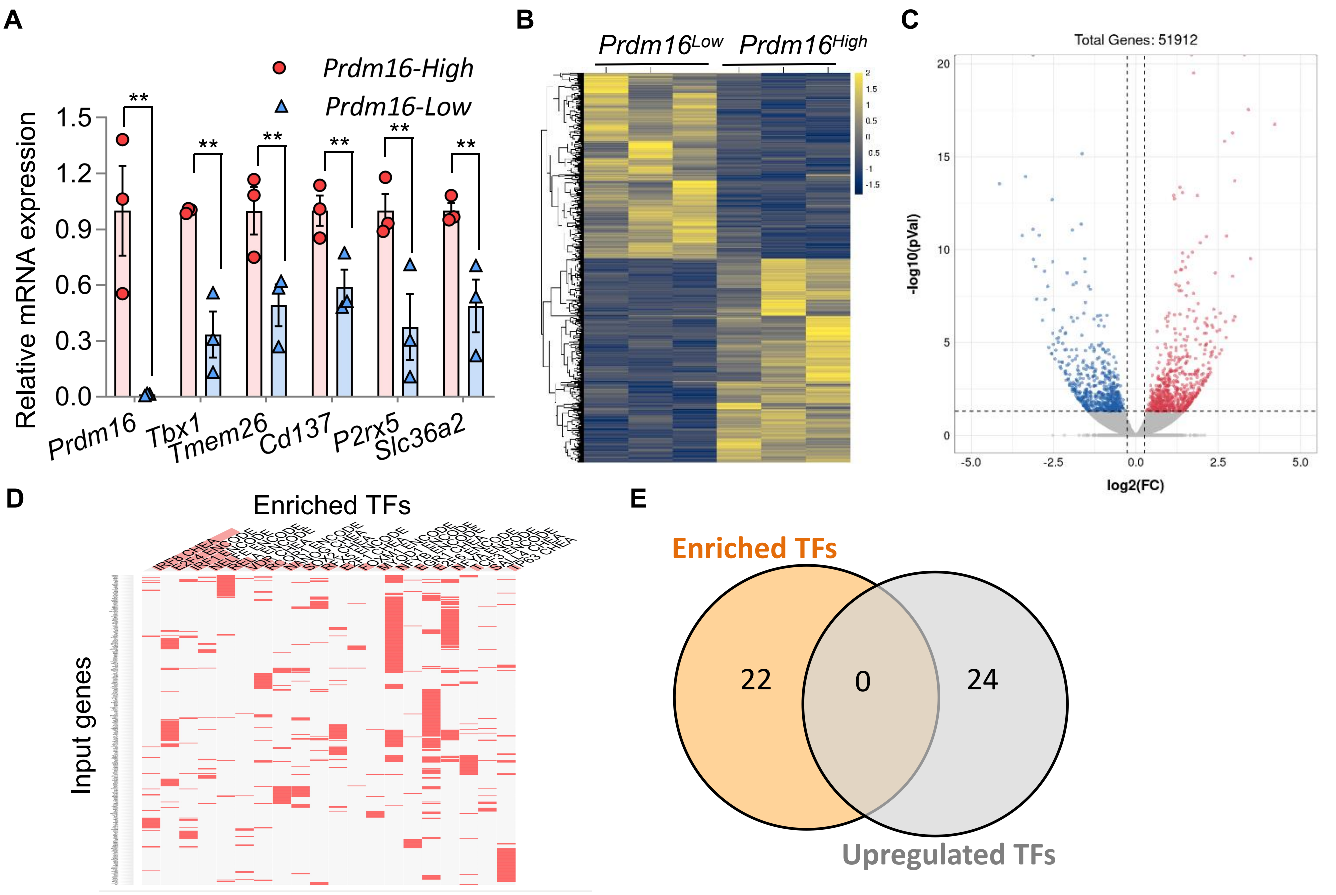

Figure S2. RNA-Seq analysis of SVC clones with high and low *Prdm16* expression.
SVC clones showing the top three highest (*Prdm16*^{High}) and lowest (*Prdm16*^{Low}) luciferase activity were subjected to RNA-Seq. (A) Relative mRNA expression in *Prdm16*^{High} and *Prdm16*^{Low} clones was examined by qPCR (n=3/group). (B) Heatmap of differentially expressed genes (DEGs). (C) Volcano plot of total genes (*Prdm16*^{High} vs. *Prdm16*^{Low}). Genes with fold change (FC) >2 or < 0.5, adjusted P value < 0.05 were set as DEGs. (D) TF enrichment analysis in upregulated DEGs using ENCODE and ChEA datasets. (E) Venn diagram of upregulated TFs and enriched consensus TFs in (D). Data are presented as mean ± SEM; statistical significances between groups were assessed by two-way ANOVA; $^{**}P < 0.01$.

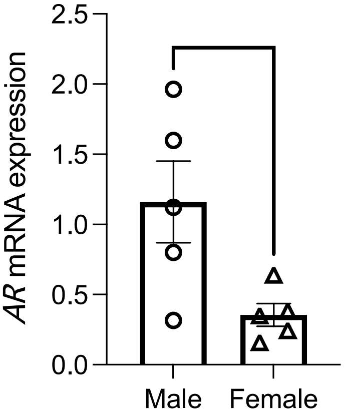

Figure S3. mRNA expression of *Ar* in inguinal white adipose tissue is sex dimorphic in mice. mRNA expression of *Ar* was determined by real time PCR in mouse inguinal white adipose tissues. (n=4/group). Data are presented as mean ± SEM; statistical significances between groups were assessed by 2-sided unpaired Student's t-test; *$P < 0.05$.

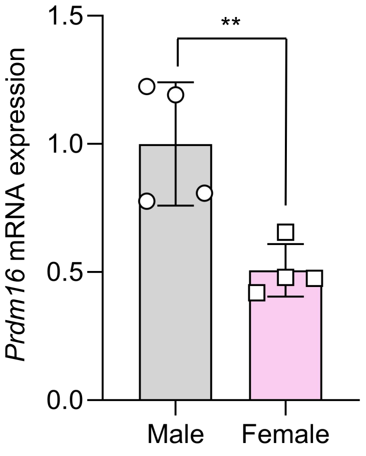

Figure S4. *Prdm16* mRNA expression is lower in female BAT.
Relative *Prdm16* mRNA expression in interscapular BAT of male and female mice (n=4/group). Data are presented as mean ± SEM; statistical significances between groups were assessed by 2-sided unpaired Student's t-test; $^{**}P < 0.01$.

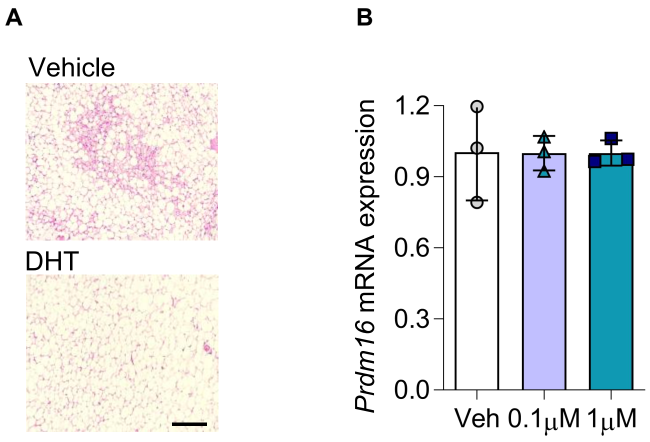

Figure S5. Androgen receptor suppresses PRDM16 expression and white adipocyte beiging.
(A) Male C57BL/6J mice were treatment with DHT or vehicle and housed at 6 ℃ for 7 days. HE staining in iWAT. Scale bar, 50 *μ*m. (B) iWAT SVCs from *Ar* KO mice were differentiated to beige adipocytes and treated with DHT for 24 hr. Relative mRNA levels of *Prdm16* was examined (n=3/group). Data are presented as mean ± SEM; statistical significances between groups were assessed by one-way ANOVA .

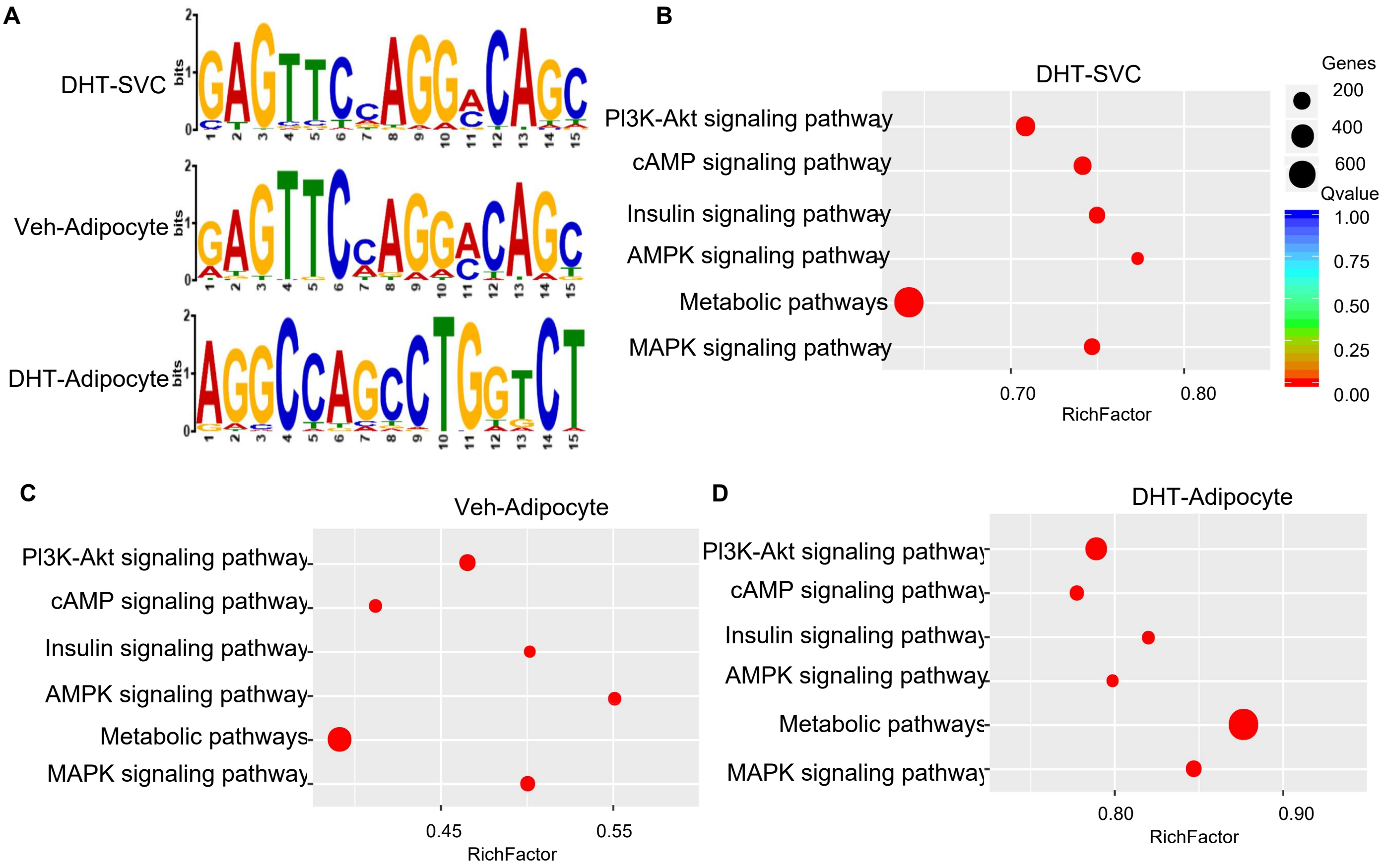

Figure S6. Motif and KEGG analysis of AR-binding events.
(A) Top consensus sequence motifs enriched at AR-binding sites using the MEME Suite tools (https://meme-suite.org/meme/). (B)-(D) KEGG pathway analysis of genes with AR enrichment in SVCs and mature adipocytes. A bar plot was generated for the enriched KEGG terms with significant Q values. RichFactor refers to the ratio of the number of genes in KEGG pathway to the number of all the annotated genes enriched in KEGG pathway

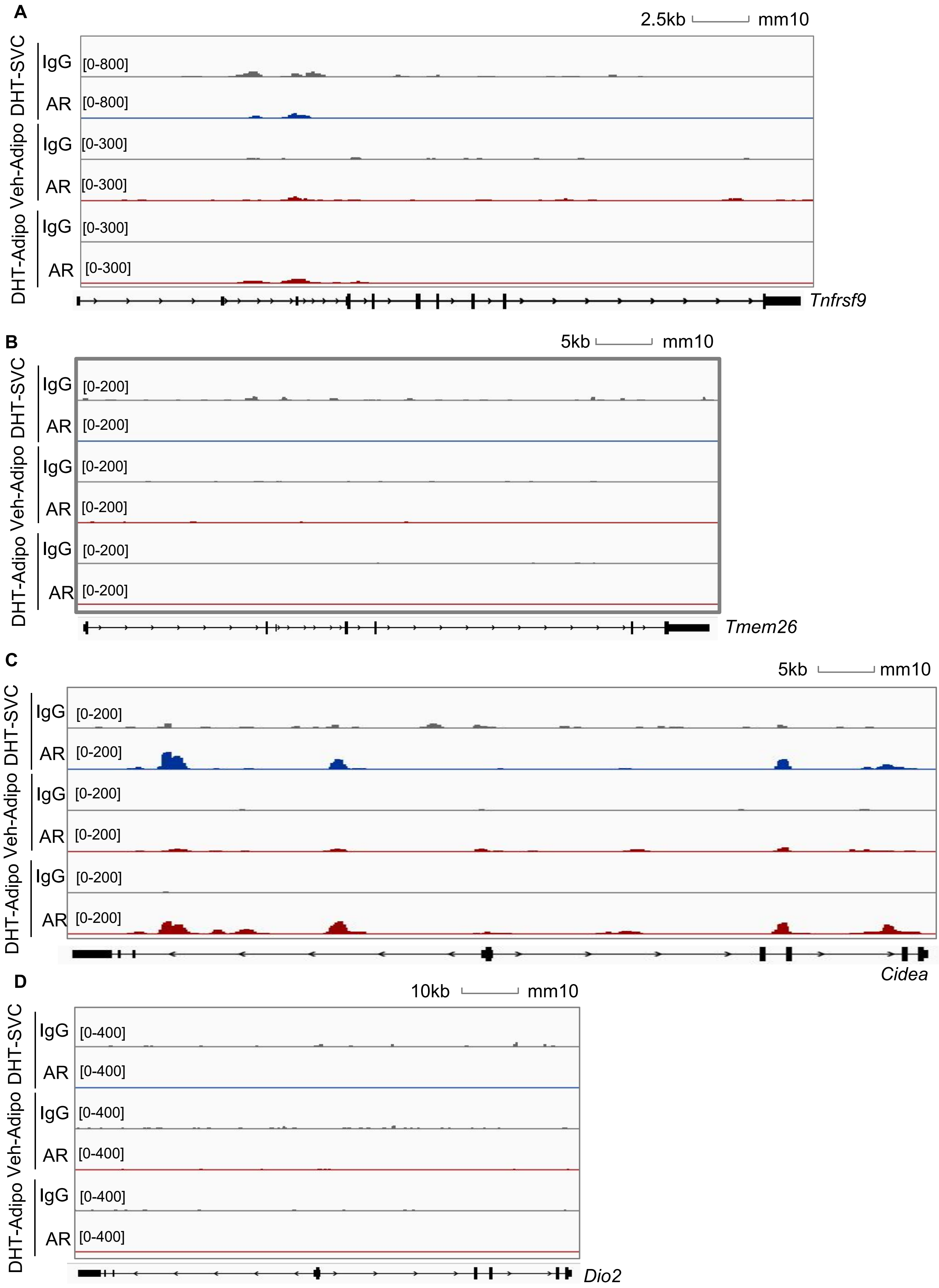

Figure S7. AR does not directly bind on other beige-related gene loci.
CUT&Tag analysis of AR-binding events in iWAT SVCs and mature adipocytes. Binding sites in beige related genes were shown. (A) *Tnfrsf9.* (B) *Tmem26.* (C) *Cidea.* (D) *Dio2.*

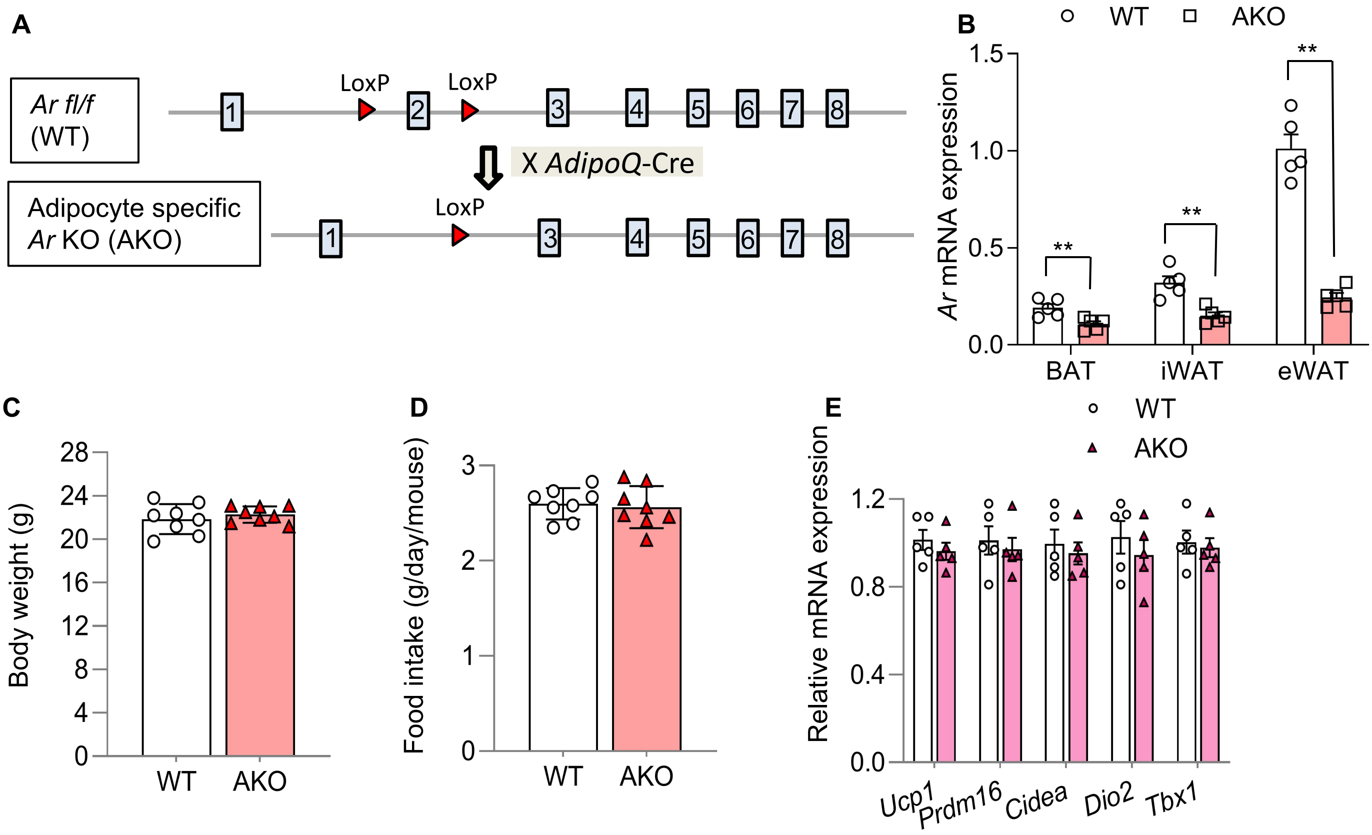

Figure S8. Generation and characterization of adipocyte-selective *Ar* KO mice (AKO).
(A) Schematic diagram of adipocyte-selective *Ar* KO mice (AKO). (B) mRNA expression of *Ar* in adipose depots of the WT and AKO mice (n=5/group). (C) Body weight and (D) food intake the WT and AKO mice (n=8/group). (E) 8-week-old male WT and AKO mice were subjected to cold exposure for 3 days. (E) qPCR analysis of the thermogenic genes in BAT of WT and AKO mice (n=5/group). Data are presented as mean ± SEM; statistical significances between groups were assessed by 2-sided unpaired Student's t-test (C)-(D) and two-way ANOVA (B) and (E); $^{**}P < 0.01$.

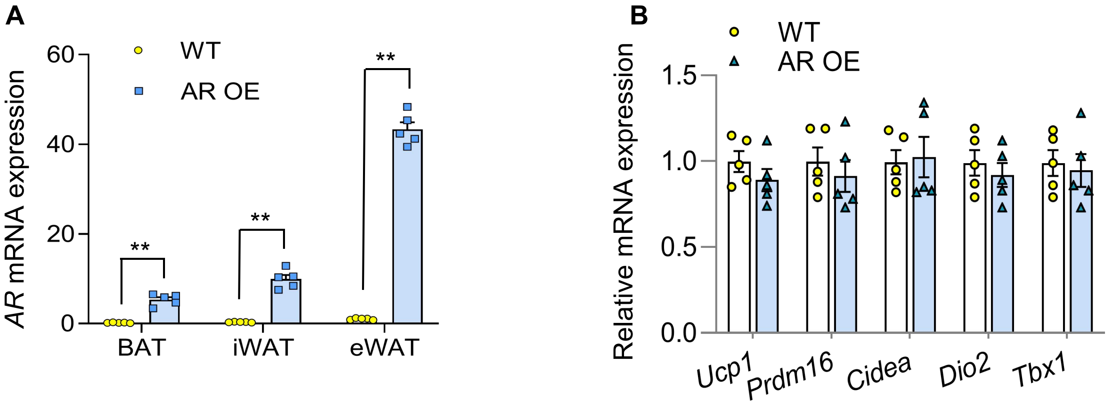

Figure S9. Generation of adipocyte specific overexpression of *AR* mice.
(A) mRNA expression of *Ar* in adipose depots of the WT and AR OE mice. (B) 8-week-old male WT and AR OE mice were housed at cold temperature for 3 days. qPCR analysis of the thermogenic genes in BAT (n=5/group). Data are presented as mean ± SEM; statistical significances between groups were assessed by two-way ANOVA; *P < 0.05, **P < 0.01.
